# Nasopharyngeal Carcinoma at the Virology Precision Oncology Nexus: Decoding Molecular Alterations for Early Intervention and Therapeutic Innovation

**DOI:** 10.3390/biom16020256

**Published:** 2026-02-05

**Authors:** Na Liu, Bin Meng, Yueshuo Li, Min Tang, Ya Cao, Li Shang, Feng Shi

**Affiliations:** 1 Key Laboratory of Carcinogenesis and Cancer Invasion of Chinese Ministry of Education, Xiangya Hospital, Central South University, Changsha 410078, China; liu_na@csu.edu.cn (N.L.); mengbin7@csu.edu.cn (B.M.); mgxxwn@csu.edu.cn (Y.L.); tangmin@csu.edu.cn (M.T.); ycao98@csu.edu.cn (Y.C.); 2 Key Laboratory of Carcinogenesis of National Health Commission, Cancer Research Institute, Xiangya School of Basic Medical Sciences, Central South University, Changsha 410078, China; 3 Molecular Imaging Research Center, Central South University, Changsha 410078, China; 4 Department of Pathology, National Clinical Research Center for Geriatric Diseases (Xiangya Hospital), Central South University, Changsha 410078, China

**Keywords:** nasopharyngeal carcinoma, Epstein–Barr virus, immunotherapy

## Abstract

Nasopharyngeal carcinoma (NPC) represents an Epstein–Barr virus (EBV)-associated malignancy showing elevated incidence in East and Southeast Asia. Early detection remains vital, as molecular abnormalities precede visible histological changes during tumor development. This review summarizes recent progress in decoding NPC’s molecular profile, including genetic mutations, epigenetic alterations, non-coding RNA networks and proteomic alterations. Importantly, these molecular discoveries are increasingly informing clinical approaches to disease management. Modern diagnostic integration of histopathology, EBV biomarkers and advanced imaging has improved detection, yet locoregional recurrence and distant metastasis remain major causes of mortality. Immunotherapy shows promising efficacy in recurrent/metastatic NPC, underscoring the potential of molecular insights to guide therapeutic innovation.

## 1. Introduction

Nasopharyngeal carcinoma (NPC) tumorigenesis involves a multistep interplay of Epstein–Barr virus (EBV) oncogenesis, genetic susceptibility, environmental carcinogens and epigenetic dysregulation [1,2,3,4,5]. Notably, NPC exhibits distinct etiological heterogeneity across geographical regions. In high-incidence areas such as East and Southeast Asia, over 95% of cases are associated with EBV infection and are classified as non-keratinizing carcinomas [1]. In contrast, in low-incidence regions (e.g., North America and Europe), a substantial proportion of NPC cases are keratinizing squamous cell carcinomas with a weaker EBV association, where tobacco and alcohol are more prominent risk factors [6]. Given that EBV-associated NPC represents the predominant form globally and offers a unique model for studying virus-driven carcinogenesis, this review will focus primarily on this subtype, while acknowledging etiological variations where relevant.

Globally, NPC incidence shows marked geographical disparity. For instance, crude incidence and mortality rates in China have been reported as 3.09 and 1.57 per 100,000, respectively [1,7,8]. Locoregional recurrence and distant metastasis remain leading causes of mortality, contributing to a five-year survival rate of 50–60% for advanced-stage (III/IV) patients [9,10]. In contrast, early-stage (I/II) patients exhibit survival rates exceeding 90%, underscoring the importance of early detection [11,12]. Molecular alterations, including genetic mutations, epigenetic dysregulation and viral oncogenic mechanisms, precede histopathological changes. These early molecular events offer promising avenues for biomarker discovery and therapeutic innovation. Traditional treatment strategies such as radiotherapy, chemotherapy and surgery are often associated with severe adverse effects and limited efficacy. In recent years, immunotherapy has emerged as a promising approach, with growing clinical evidence supporting its safety and efficacy in NPC, particularly in EBV-associated cases.

This review aims to systematically decode the molecular landscape of NPC from a virology-oncology perspective, with an emphasis on EBV-driven pathways. The first objective is to identify early molecular alterations that could aid non-invasive detection and risk stratification. The second objective is to examine how these molecular insights can guide novel treatments. Based on this framework, we summarize recent advances in early detection and therapeutic innovation derived from this integrated understanding.

## 2. EBV Infection Mechanisms and Molecular Pathogenesis

NPC is an infection-associated malignancy primarily driven by EBV. EBV, the first identified human tumor virus, is strongly implicated in the etiology of multiple lymphoid and epithelial cancers [13]. B lymphocytes and epithelial cells are the main target cells of EBV. The lifecycle of EBV consists of two stages, the lytic cycle and the latent phase. Upon primary infection, EBV typically establishes a persistent, lifelong latent infection in memory B cells [13]. In addition to its role in B-cell persistence, EBV is also directly associated with epithelial cancers, most notably NPC and a subset of gastric cancers. Histologically, NPC is classified into keratinizing squamous cell carcinoma, differentiated non-keratinizing carcinoma, undifferentiated non-keratinizing carcinoma, and basaloid squamous cell carcinoma. Notably, EBV is detected in nearly all cases of undifferentiated NPC, particularly in endemic areas [1,14]. During EBV infection of epithelial cells, the viral BMRF2 protein first anchors to integrin. Subsequently, the gH/gL heterodimer cooperatively binds to both integrin and ephrin receptor A2. This binding triggers conformational activation of the fusion protein gB, which ultimately mediates the fusion of the viral envelope with the host cell plasma membrane [15,16]. Thus, the EBV envelope glycoproteins gH/gL and gB are critical mediators of EBV infection in epithelial cells. And gH/gL and gB represent potential targets for prophylactic vaccine development [17].

EBV exhibits three distinct latency programs (types I, II and III), defined by the specific repertoire of viral latent genes expressed. Type III latency (e.g., in post-transplant lymphoproliferative disease) involves expression of all EBNA proteins, LMP1 and LMP2 [16]. NPC is classically associated with type II latency, which is characterized by the expression of EBNA1, LMP1 and LMP2A/B, along with abundant non-coding RNAs such as BARTs and EBERs [16]. These type II latency molecules are not only central to oncogenesis but also serve as foundational targets for diagnostic biomarkers and therapeutic strategies. LMP1 acts as a constitutively active mimic of the tumor necrosis factor receptor (TNFR), hijacking multiple signaling pathways (including NF-κB, JAK/STAT and MAPK) to promote cell survival, proliferation and inflammation [16,18]. LMP1 is also detectable by immunohistochemistry, aiding clinical diagnosis [19]. LMP2A mimics B-cell receptor signaling and can enhance epithelial cell motility and survival. EBNA1 is essential for viral genome maintenance and also modulates host cell transcription and stability [16,18]. EBNA1 is a key target for both serological diagnosis and therapeutic vaccines [20,21]. Together with the non-coding RNAs, these viral products collectively disrupt key cellular homeostasis and drive malignant transformation through sustained proliferation, avoidance of immune detection, and inhibition of apoptosis [22]. However, as NPC progresses, lytic genes begin to be expressed. The immediate-early gene BZLF1 (encoding the Zta protein) serves as the master switch for initiating the lytic cycle [23]. In NPC, focal and abortive expression of BZLF1 is common and biologically active [23,24]. Detection of BZLF1 in tissues is associated with enhanced tumor metastasis and invasion, angiogenesis, resistance to cell death and immune evasion [23,24,25].

The high prevalence of EBV in this subtype underscores its central role in oncogenesis. Given its strong epidemiological association with NPC, EBV-derived biomarkers have become cornerstone tools for screening and diagnosis. Detections targeting EBV DNA and EBV antigens (anti-VCA IgA, anti-EBNA1-lgA and anti-EA IgG) are widely used for NPC screening in high-risk populations [26]. Additionally, anti-BZLF1 (anti-Zta) IgA reflects chronic EBV lytic activity and shows improved diagnostic sensitivity compared to VCA-IgA alone [27].

## 3. Current Diagnostic Landscape

**Figure 1:**
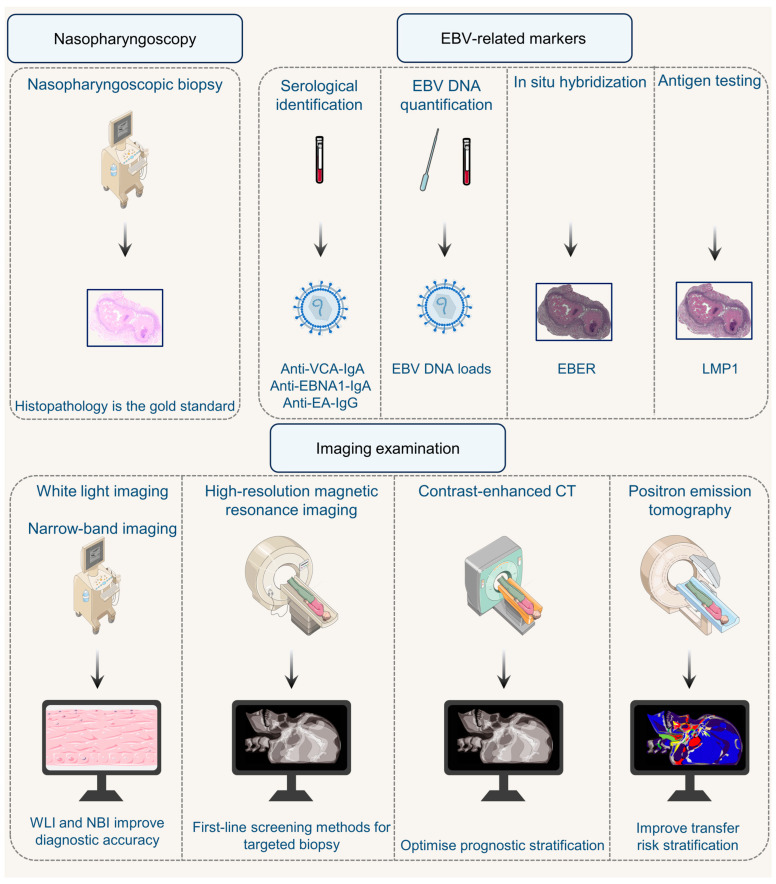
Diagnostic framework of NPC. Nasopharyngoscopy with biopsy of suspicious lesions remains the cornerstone of pathological confirmation. Advanced endoscopic techniques, including WLI and NBI, enhance the visualization of mucosal abnormalities and guide targeted biopsies. Imaging modalities such as high-resolution MRI, PET/CT and CE-CT provide critical adjunctive value for local and systemic staging, prognostic stratification, and surveillance. Given the strong etiological association of NPC with EBV infection, virological biomarkers play a central role. EBV DNA load and EBV-specific serological antibodies are pivotal for early screening, risk stratification and post-treatment monitoring. At the tissue level, in situ hybridization for EBER is the gold standard for confirming EBV association within tumor cells, complemented by immunohistochemical detection of LMP1.

Currently, the diagnosis of NPC relies on a multimodal and integrated system combining clinical examination, imaging, molecular biology and digital technologies (Figure 1).

### 3.1. Standard Diagnostic Protocol and Routine Clinical Practice

International guidelines, such as the ESMO-EURACAN Clinical Practice Guidelines, recommend a standardized multimodal protocol for NPC diagnosis. The gold standard for definitive diagnosis is endoscopic-guided biopsy of suspicious primary nasopharyngeal lesions [28]. High-resolution magnetic resonance imaging (MRI) and narrow-band imaging (NBI) serve as first-line investigations for detecting subtle mucosal abnormalities and guiding targeted biopsies in early-stage lesions that evade conventional endoscopic visualization [29,30,31]. However, MRI faces diagnostic challenges in distinguishing T1-stage NPC from benign hyperplasia and in identifying diffuse, symmetrical tumors that lack focal masses. Consequently, endoscopic correlation is often necessary, even though MRI demonstrates higher sensitivity than endoscopic examination alone [32]. Positron emission tomography/computed tomography (PET/CT) has demonstrated established feasibility and efficacy in tumor diagnosis, treatment planning, prognostic evaluation and disease surveillance. Several radiotracers, including fluciclovine F-18, gallium Ga-68 DOTATATE and lutetium Lu-177 DOTATATE, are FDA-approved for PET imaging [33]. At the tissue level, in situ hybridization for EBERs remains the gold standard for confirming EBV association, demonstrating near-universal positivity in NPC specimens [18,28]. For staging and risk assessment, routine work-up includes medical history, physical examination (including cranial nerves), complete blood count, serum biochemistry, nasopharyngoscopy and imaging [28]. MRI is the most accurate method for defining local and nodal tumor staging and should be the preferred imaging modality [28]. FDG-PET/CT provides further accuracy in nodal staging [28].

### 3.2. Augmentation by Advanced Imaging and Digital Pathology

The clinical application of artificial intelligence (AI) in NPC diagnosis is context-dependent, addressing specific challenges across different clinical scenarios. Specifically, a Siamese deep convolutional neural network integrating both white light (WLI) and NBI endoscopy images aids in distinguishing early-stage NPC from benign lesions, achieving high diagnostic accuracy [34]. This approach serves as a crucial computer-aided tool for clarifying ambiguous cases in screening. In histopathological diagnosis, AI-driven digital pathology tools primarily address workflow efficiency. Whole slide imaging enables AI analysis, reducing diagnostic time burdens [35]. Weakly supervised models, such as the Tokens-to-Token Vision Transformer (WS-T2T-ViT), are significant for settings with limited expert annotation resources. These models achieve high diagnostic accuracy on whole slide images using only slide-level labels, ensuring robust performance while minimizing labor [36].

For treatment stratification in locoregionally advanced NPC, contrast-enhanced CT radiomics provides functional prognostic information beyond anatomical TNM staging. A radiomics-based model integrating specific image features and N stage can identify patients with a favorable prognosis, thus helping to select candidates who may be suitable for deintensified therapy [37]. In post-treatment surveillance for recurrence, AI augmentation of MRI improves specificity. An AI-aided MRI model demonstrates performance approaching that of PET/CT in select cohorts, offering a potentially more accessible and cost-effective monitoring strategy for detecting local recurrence [38].

### 3.3. Integration of Molecular and Virological Biomarkers

Molecular alterations in premalignant lesions often precede morphological changes. highlighting the potential of molecular biomarkers for early detection and precision classification. For NPC, which is strongly associated with EBV infection, EBV-related biomarkers are crucial for early detection, risk stratification and monitoring [39]. Clinically validated markers include plasma EBV DNA load and serological antibodies (e.g., anti-VCA IgA, anti-EBNA1-IgA, anti-EA IgG) [1,40,41]. However, their application differs based on clinical context. Beyond viral load and serology, specific genetic variations in EBV also hold diagnostic and prognostic value. For instance, evolutionary analysis of the LMP1 gene, a key viral oncogene, has identified genetic variants associated with increased oncogenic potential. Certain LMP1 mutants are linked to a higher metastasis potential, providing a molecular layer for understanding tumor aggressiveness [42].

In population screening for early detection, serological antibodies are pivotal. A landmark trial demonstrated that combining VCA-IgA and EBNA1-IgA serology improved early NPC diagnosis rates and reduced NPC-related mortality [43,44]. For individuals with positive serology or high risk, confirmatory testing often involves plasma EBV DNA due to its higher specificity in resolving false positives [9,41,45]. Nasopharyngeal brushing offers a direct sampling alternative. Notably, for non-invasive screening, EBV DNA methylation analysis in nasopharyngeal brushing samples shows superior accuracy to DNA load quantification and avoids endoscopy [46].

In post-diagnosis surveillance and prognosis, plasma EBV DNA is the cornerstone biomarker [9,47,48]. Specifically, post-treatment persistence of EBV DNA correlates with poorer survival outcomes [49]. For detecting recurrence during follow-up, plasma EBV DNA demonstrates high sensitivity, particularly for distant metastasis [50]. Compared to seromarkers like EA-IgA, which also predicts survival, plasma EBV DNA provides a more immediate reflection of tumor burden and is superior to PBMC-based assays for monitoring [51,52].

These biomarkers are integrated into clinical algorithms based on purpose. Serology is preferred for initial mass screening. Plasma EBV DNA is central for confirming screen-positive cases, staging, prognostic stratification, and monitoring treatment response and recurrence. Emerging multi-analyte models combining these markers promise enhanced risk stratification.

## 4. Molecular Regulatory Networks of Nasopharyngeal Cancer

NPC pathogenesis arises from multifaceted molecular changes (Table 1). These changes include genetic mutations, epigenetic modifications and non-coding RNA/protein dysregulation. A comprehensive understanding of these mechanisms is crucial. However, the translational potential of these findings depends on rigorous validation and a clear understanding of their clinical applicability.

### 4.1. Genetic Aberrations

**Figure 2:**
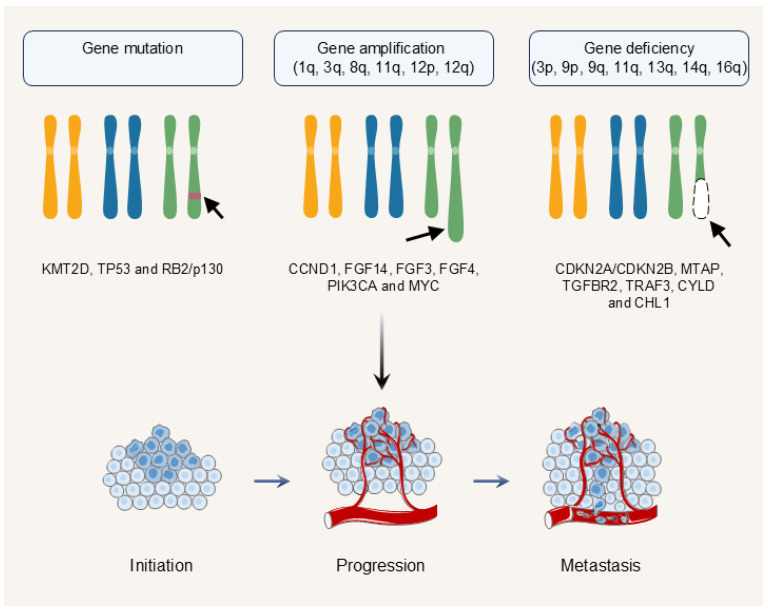
Genetic and Molecular Drivers of NPC Pathogenesis. NPC exhibits a relatively low mutation rate with frequent copy number alterations. The disease progression is driven by cumulative genetic alterations that disrupt tumor suppression mechanisms, activate oncogenic signaling pathways, and enhance immune evasion capabilities. KMT2D, TP53, and RB2/p130 are the most frequently mutated genes. Whole-genome sequencing identified recurrent chromosomal imbalances, including frequent gains at 1q, 3q, 8q, 11q, 12p and 12q, with key amplified oncogenes (CCND1, FGF14, FGF3, FGF4, PIK3CA and MYC). High-frequency allelic losses occur at 3p, 9p, 9q, 11q, 13q, 14q, and 16q, involving critical tumor suppressor genes (CDKN2A/CDKN2B, MTAP, TGFBR2, TRAF3, CYLD and CHL1).

NPC development involves cumulative genetic changes (Figure 2). This cancer has a relatively low mutation rate but frequent copy number alterations. Targeted next-generation sequencing of 40 primary NPC tumors identified recurrent mutations. The most frequently mutated genes included *KMT2D*, *CYLD* and *TP53* [53]. This was a retrospective study using a targeted 450-gene panel. Its statistical power to define prevalence is limited by the small sample size. A separate study found mutations in the *RB2/p130* (exons 19–22) gene in 30% of primary NPC tumors from a North African cohort [54]. This is an exploratory finding from a small, region-specific sample. Its generalizability is unclear and requires validation in larger, diverse cohorts. Whole-genome studies show recurrent chromosomal gains at 1q, 3q, 8q, 11q, 12p and 12q, and losses at 3p, 9p, 9q, 11q, 13q, 14q and 16q [55,56,57,58,59]. These alterations are not equal in functional importance. Some are likely passenger events, while others are drivers. Key amplified regions include 11q13.1–13.3 driving *CCND1* overexpression [60]. *CCND1* knockdown suppresses NPC cell proliferation [60]. Critically, 11q13 amplification is linked to clinical outcome. In a phase II trial, none of the 12 patients with this alteration responded to the anti-PD-1 therapy toripalimab [57]. This highlights a potential genetic determinant of immune resistance. *PIK3CA* amplification is found in 21.6% of cases in a Tunisian cohort [61]. This retrospective study of 88 patients found a strong association between PIK3CA amplification and advanced disease features, including metastasis and reduced overall survival. *MYC* (8q24) is another commonly amplified oncogene [58]. Frequent deletions also occur. Homozygous deletions at 9p21.3 (*CDKN2A*/*CDKN2B* and *MTAP*) and losses of *TGFBR2*, *TRAF3* and *CYLD* drive NF-κB activation and immune evasion [62]. *CHL1* (3p26.3) is downregulated in most tumors, and its re-expression suppresses growth [63]. In summary, NPC progression is driven by a combination of genetic events that disrupt tumor suppression, activate oncogenic pathways such as PI3K and MYC, and enhance immune evasion. The association between 11q13 amplification and immunotherapy resistance is a compelling example with direct clinical relevance.

### 4.2. Epigenetic Dysregulation

DNA methylation is a pivotal epigenetic mechanism in NPC [64,65]. Aberrant promoter methylation silences tumor suppressor genes and activates oncogenes. Hypermethylation silences genes like *NFAT1*, *ACAT1* and *USP44* and *HOPX* [66,67,68]. Hypomethylation activates genes like *FGF5*, *ELF3* and *S100A4* [69,70,71]. These changes promote proliferation, metastasis and therapy resistance. Methylation markers show promise for diagnosis. Multi-gene panels in tissue show high diagnostic specificity but variable sensitivity (26–66%) [72]. Liquid biopsy approaches are increasingly promising for non-invasive detection. For example, *RERG*/*ZNF671* methylation in circulating cell-free DNA (ccfDNA) showed high diagnostic accuracy [73]. A four-gene panel (*RASSF1A*, *WIF1*, *DAPK1*, *RARβ2*) in plasma and nasopharyngeal brushing improved early detection when combined with EBV DNA testing [74]. Blind brushing combining EBV-BILF2 and host *IMPA2* methylation achieved 84.62% sensitivity and 98.44% specificity [75]. *SEPT9* methylation in nasopharyngeal swabs is also a potential early biomarker [76]. However, most studies are exploratory and retrospective. Their clinical utility requires validation in larger, prospective cohorts. Methylation also has prognostic value. Hypermethylation of *WIF1*, *UCHL1*, *RASSF1A*, *CCNA1*, *TP73* and *SFRP1* correlates with poorer survival [77]. *HOPX* hypermethylation predicts advanced stage and poor outcome [78]. Despite strong potential, key challenges for clinical implementation remain. These include assay standardization, defining optimal biomarker panels, and integrating tests into existing screening workflows.

### 4.3. MicroRNA Dysregulation

MicroRNAs (miRNAs) regulate gene expression post-transcriptionally [79,80,81]. In NPC, EBV-encoded and host miRNAs interact, promoting tumorigenesis and immune evasion [82,83,84]. A study analyzed serum from 208 NPC patients and 238 healthy controls. It reported a 5-miRNA panel (let-7b-5p, miR-140-3p, miR-192-5p, miR-223-3p and miR-24-3p) with high diagnostic accuracy [85]. The AUC values were 0.910, 0.916 and 0.968 across training, testing and external validation stages, respectively [85]. A 4-miRNA (miR-22, miR-572, miR-638 and miR-1234) signature predicted survival [86]. Plasma EBV-miR-BART8-3p levels correlate with reduced survival [87]. Platelet-absorbed miR-34c-3p and miR-18a-5p showed high diagnostic accuracy (combined AUC = 0.954) [88]. Salivary miRNAs represent a promising non-invasive source of biomarkers. A pilot microarray study on saliva from 22 NPC patients and 25 healthy controls identified a signature of 12 down-regulated miRNAs (miR-30b-3p, miR-575, miR-650, miR-937-5p, miR-1202, miR-1203, miR-1321, miR-3612, miR-3714, miR-4259, miR-4478 and miR-4730) with an exceptional reported AUC of 0.999 [89]. However, such exceptionally high performance requires critical evaluation. Factors like sample size, population heterogeneity and the need for independent validation are important limitations. Therapeutically, strategies aim to suppress oncogenic miRNAs (miR-28-3p or EBV-miR-BART22) or restore tumor-suppressive ones (miR-205 or miR-873) [83,90,91,92]. Importantly, miRNA networks do not operate in isolation. Their dysregulation is often downstream of genetic and epigenetic alterations, such as the methylation-mediated silencing of miRNA host genes. Future work should integrate these layers to define unified regulatory circuits.

### 4.4. Long Non-Coding RNA Alterations

Long non-coding RNAs (lncRNAs) are transcripts exceeding 200 nucleotides in length [93]. They function as competing endogenous RNAs (ceRNAs) or activating RNAs [94]. For instance, FAM225A promotes NPC tumorigenesis and metastasis by sponging miR-590-3p and miR-1275, leading to the activation of ITGB3 and the FAK/PI3K/AKT pathway [95]. Similarly, the EBV-upregulated lncRNA BC200 acts as a ceRNA for miR-6834-5p, resulting in increased expression of the TYMS, which is implicated in cancer progression [96]. A prominent functional theme among NPC-associated lncRNAs is the mediation of radiation resistance, a major clinical challenge. A study analyzing 220 formalin-fixed NPC specimens retrospectively linked high RRFERV expression to poor clinical outcomes [97]. Mechanistically, it acts as a ceRNA, sponging miR-615-5p and miR-1293 to stabilize TEAD1, thereby promoting radioresistance [97]. Similarly, PVT1 and HOTAIRM1 have been reported to drive radioresistance through pathways involving DNA repair and m6A modification, respectively [98,99]. While these mechanistic discoveries are rich, the clinical translation of lncRNAs as biomarkers or therapeutic targets remains preliminary. Some studies have proposed circulating lncRNAs like POU3F3, MALAT1, AFAP1-AS1 and AL359062 as potential diagnostic or prognostic biomarkers [100,101]. However, many of these findings are exploratory, often derived from single-center, retrospective studies with limited sample sizes and lacking independent validation cohorts. A major challenge for their clinical application is the typically low abundance of many lncRNAs in circulation, coupled with the complexity of their regulatory networks, which complicates reliable detection and therapeutic targeting.

### 4.5. Proteomic Alterations

Proteomic studies bridge genomic alterations with functional phenotypes and offer a direct route for biomarker discovery. However, the clinical translation of most findings remains at an exploratory stage, pending rigorous validation in large-scale, prospective cohorts. Secretome analysis identified plasma CCL5 as a diagnostic biomarker (AUC = 0.801), but this finding was based on a limited cohort and has not been widely adopted in clinical practice [102]. A serum study identified HSP70, sICAM-1 and SAA as potential metastasis markers [103]. These proposed biomarkers originate from single-center studies with limited sample sizes and lack clear demonstration of superior diagnostic advantage over established standards. Early studies using 2D electrophoresis and mass spectrometry identified differentially expressed proteins like stathmin, 14-3-3sigma and annexin I in NPC tissues, with their expression levels related to metastatic potential [104]. However, these early findings currently lack independent validation. The most significant translational progress involves combining proteomic markers with the established gold standard. Serum exosomal cyclophilin A combined with EBV-VCA-IgA improved diagnostic specificity [105]. Combining plasma EBV DNA load with serum C-reactive protein improved prognosis in advanced cases [106]. These studies highlight the biomarker discovery value of proteomics. However, few proposed protein biomarkers offer a clear advantage over established markers like EBV DNA. Proteomics also clarifies host–virus interactions. iTRAQ-based profiling identified 12 upregulated proteins (VDAC1, S100-A2, Hip-70, Ubiquitin, TPT1-like protein, 4F2hc, Keratin-75, TB8, Dynein light chain 1, LDH-B, TIM and HMG-1) in EBV-infected cells [107]. The proteomic landscape of NPC is richly characterized at a discovery level. However, the proposed protein biomarkers generally lack the extensive multi-center validation and proven clinical utility.

## 5. Immunotherapy for Nasopharyngeal Carcinoma: Bridging Molecular Insights and Clinical Translation

More than 70% of NPC patients present with stage III/IV disease at initial diagnosis due to the nasopharynx’s concealed anatomy and nonspecific early symptoms [108]. While standard chemoradiotherapy remains foundational, 20–30% of patients experience recurrence, highlighting the need for novel strategies [109,110]. The profound molecular underpinnings of NPC, particularly its universal association with EBV, directly create a rationale for immunotherapy (Table 2). EBV-driven oncogenesis establishes a unique tumor microenvironment (TME) characterized by immune evasion mechanisms, which these therapies aim to reverse.

### 5.1. Adoptive T-Cell Therapy: Reconstituting Antiviral Immunity

Adoptive T-cell therapy (ACT) aims to compensate for the immune system’s inability to adequately control latent EBV infection. This approach involves ex vivo expansion and infusion of cytotoxic T lymphocytes (CTLs) specific for EBV latent cycle antigens (EBNA1, LMP1, LMP2) [111,112,113]. Clinical studies report disease control in a subset of patients with refractory NPC, validating the concept of targeting these viral oncoproteins [111,112,113]. However, the tumor microenvironment can resist ACT through mechanisms including upregulation of immune checkpoints and secretion of immunosuppressive cytokines.

### 5.2. Immune Checkpoint Blockade: Targeting Virus and Epigenetically Induced Immunosuppression

A primary molecular feature enabling immunotherapy in NPC is the high tumor cell expression of programmed death-ligand 1 (PD-L1), observed in over 80% of cases [109,114]. This expression is not stochastic but is orchestrated by EBV through a multi-layer regulatory network involving the key oncoprotein LMP1 and immune-modulatory miRNAs like miR-BART11 and miR-BART17-3p [82,115]. This molecular backdrop justifies the clinical efficacy of PD-1 inhibitors (toripalimab, camrelizumab, tislelizumab, sintilimab) combined with chemotherapy, as established in phase 3 trials for recurrent/metastatic NPC [109,114,116,117]. However, intrinsic and acquired resistance limits monotherapy efficacy. Resistance mechanisms may involve compensatory upregulation of alternative checkpoints, such as lymphocyte-activation gene 3 (LAG-3) or Cytotoxic T-lymphocyte antigen 4 (CTLA-4) [118,119,120]. Consequently, combination strategies targeting multiple pathways are critical. Dual PD-1/CTLA-4 blockade (e.g., nivolumab/ipilimumab) shows enhanced efficacy, potentially by overcoming T-cell exhaustion states reinforced by the co-expression of these checkpoints [121]. The clinical activity of bispecific antibodies targeting PD-1 and CTLA-4, such as cadonilimab and QL1706, further supports the mechanistic synergy of co-inhibitory pathway blockade [122,123]. Emerging targeting of LAG-3, as with LBL-007 combined with toripalimab, represents a logical extension of this principle, aiming to restore the function of T-cell populations [124].

### 5.3. EBV-Targeted Strategies: From Prophylaxis to Therapeutic Vaccination

Given the etiological role of EBV, therapeutic strategies directly targeting viral antigens are a paradigm of translational innovation rooted in molecular virology. Prophylactic vaccine development focuses on glycoproteins gH/gL and gB, which are critical for epithelial cell entry. Nanoparticle vaccines displaying these antigens, such as gH/gL NPs or gB-I53-50 NPs, are designed based on structural virology to elicit potent neutralizing antibodies, showing protective efficacy in preclinical models [17,125]. For therapeutic vaccination in established NPC, the target shifts to latent phase antigens. The choice of EBNA1 and LMP2 as targets is mechanistically informed. EBNA1 is essential for viral genome maintenance and is a dominant CD4^+^ T-cell target; LMP2 is expressed in type II latency and provides epitopes for CD8^+^ T cells [21,126]. Clinical trials of vaccines like MVA-EL (encoding EBNA1/LMP2) demonstrate the expansion of specific T-cell populations, confirming immune priming [127,128]. A key challenge for therapeutic efficacy is the relatively low and heterogeneous expression of these target antigens in tumor cells, which can be further downregulated by epigenetic mechanisms, allowing immune escape.

**Table 2:** Progress in immunotherapy strategies.

Treatment Strategy	Setting/Trial Identifier	Phase	Treatment	Sample	Sample Size	Efficacy	Reference
Immune checkpoint blockade	NCT03581786 (JUPITER-02)	Phase III	Toripalimab + chemotherapy	Recurrent/metastatic NPC	289	Median PFS: 21.4 months; ORR: 78.8%; DCR: 88.4%; Median DOR: 18.0 months	[114]
	NCT03707509 (CAPTAIN-1st)	Phase III	Camrelizumab + chemotherapy	Recurrent/metastatic NPC	263	ORR: 87.3%; DCR: 96.3%	[116]
	NCT03924986 (RATIONALE-309)	Phase III	Tislelizumab + chemotherapy	Recurrent/metastatic NPC	263	ORR: 69.5%; DCR: 89.3%; Median DOR: 8.5 months	[117]
	NCT03700476 (CONTINUUM)	Phase III	Sintilimab + chemotherapy	Locoregionally advanced NPC patients (stage III–IVa)	425	EFS rate at 36 months: 86%; DMFS rate at 36 months: 90%; LRFS rate at 36 months: 93%; OS rate at 36 months: 92%	[109]
	NCT03097939	Phase II	Nivolumab + ipilimumab	Recurrent/metastatic EBV-associated NPC	40	PR: 37.5%; SD: 17.5%; PD: 42.5%; DCR: 55%	[121]
	ChiCTR2200067057	Phase II	Cadonilimab (PD-1/CTLA-4 bispecific) + chemotherapy	Anti-PD-1-resistant recurrent/metastatic NPC	25	ORR: 68%; DCR: 92%; CR: 12%; PR: 56%; SD: 24%; PD: 4%	[122]
	NCT04296994 and NCT05171790	Phase I/Ib	QL1706 (PD-1/CTLA-4 bispecific)	NPC	110	CR: 0%; PR: 24.5%; SD: 24.5%; PD: 47.3%; ORR: 24.5%; DCR: 49.1%	[123]
	NCT05102006	Phase Ib/II	LBL-007 (LAG-3) + toripalimab	Advanced NPC	30	PR: 33.3%; SD: 41.7%; PD: 25.0%; ORR: 33.3%; DCR: 75.0%	[124]
EBV-directed vaccination		Preclinical	gH/gL nanoparticle vaccine	Humanized mice		Induced neutralizing antibodies; prevented lethal EBV challenge	[17]
		Preclinical	gB nanoparticle vaccine	Mice/non-human primate		Induced neutralizing antibodies; prevented lethal EBV challenge	[125]
	NCT01256853	Phase I	MVA-EL (encode an EBNA1/LMP2 fusion protein)	EBV-positive NPC	18	Enhanced T-cell responses in 15/18 patients	[127]
	NCT01147991	Phase I	MVA-EL (encode an EBNA1/LMP2 fusion protein)	EBV-positive NPC	14	Enhanced T-cell responses in 8/14 patients	[128]
Adoptive T-cell therapy		Clinical trial	Autologous EBV-specific CTLs	Stage IV NPC	10	Induces LMP-2 specific immune responses	[111]

## 6. Conclusions and Future Directions

NPC remains a significant clinical challenge. Its etiology involves a complex interplay of EBV infection, genetic susceptibility and environmental factors. Over recent decades, research has successfully decoded the molecular landscape of this disease. This includes genetic mutations, epigenetic reprogramming and non-coding RNA dysregulation. These discoveries have directly informed clinical practice. They have improved early detection through advanced EBV biomarker profiling. They have also guided the development of novel immunotherapies. Looking forward, the integration of emerging technologies promises to deepen our understanding. It will also enable more precise and personalized management of NPC.

Current diagnostic paradigms effectively integrate histopathology, EBV biomarkers and advanced imaging. Future refinements will focus on enhancing accuracy and accessibility. AI models are being developed to interpret endoscopic and radiological images. These models can improve the identification of early-stage tumors and assist in treatment planning. For example, an AI-powered endoscopic analysis system trained on thousands of images significantly improved diagnostic accuracy [129]. Furthermore, AI-based radiomics extracts subtle features from medical images for prognosis prediction and recurrence monitoring [130]. The next generation of diagnostic platforms will integrate these AI tools with molecular data streams. This integration will enable more precise and dynamic patient assessment.

A deep understanding of molecular regulatory networks is fundamental for advancing therapy. NPC pathogenesis involves genetic, epigenetic, and non-coding RNA alterations. Key genetic events like 11q13 amplification are linked to immunotherapy resistance. Epigenetic changes such as DNA methylation silence tumor suppressor genes. Non-coding RNAs, including both EBV-encoded and host miRNAs, are central to immune evasion. These interconnected layers of regulation drive tumor progression and treatment failure. Future research must continue to decipher these complex networks to identify new vulnerabilities.

Prevention and therapy are becoming increasingly targeted, guided by molecular insights. Immunotherapy is now a cornerstone for recurrent or metastatic NPC. The efficacy of PD-1 inhibitors combined with chemotherapy is established. However, resistance remains a challenge. Future strategies involve rational combination therapies, such as dual blockade of PD-1 with CTLA-4 or LAG-3, to overcome resistance. Adoptive T-cell therapy infusing EBV-specific T cells has shown disease control in refractory patients. EBV-targeted vaccine strategies span from prevention to treatment. Prophylactic vaccines aim to block viral entry. Therapeutic vaccines target latent antigens in tumor cells. A key ongoing challenge is the low and heterogeneous expression of these target antigens.

Future research in NPC will be transformed by advanced molecular profiling technologies. Spatial transcriptomics is a key emerging tool. This technology can map the precise location of specific cell populations and molecular pathways. For instance, a recent study combined single-cell and spatial transcriptomics. It revealed the molecular and histological mechanisms underlying radio-resistance and immune escape in recurrent NPC [131]. This approach can identify distinct tumor subregions. It can also clarify stromal-immune cell interactions. These are crucial for understanding treatment failure. Single-cell multi-omics goes a step further. It enables simultaneous profiling of the genome, transcriptome, epigenome, and proteome within individual cells. Applying these technologies to premalignant lesions and paired primary-metastatic samples is a clear future direction. It will uncover the cellular origins of NPC and the dynamics of metastatic evolution.

In conclusion, the field of NPC research is at a pivotal point. The traditional approach is giving way to a new paradigm. This paradigm is based on spatial context, single-cell resolution, and computational data integration. By embracing these technologies, future research will bridge the gap between molecular discovery and clinical application. This will enable true precision medicine. It promises improved risk prediction, earlier diagnosis, and personalized treatment strategies for every NPC patient.

## Figures and Tables

**Table 1:** Key oncogenic mechanisms in nasopharyngeal carcinoma.

Core Functional Theme	Genetic Alterations	Epigenetic Alterations	Non-Coding RNA Alterations	Proteomic Alterations
Cell proliferation	*CHL1* downregulation; *MYC* amplification	*ACAT1* hypermethylation-mediated silencing	miR-106a-5p upregulation; FAM225A and BC200 upregulation	
Metastasis and invasion	*PIK3CA* amplification	*NFAT1* and *ACAT1* hypermethylation-mediated silencing; *FGF5* and *S100A4* hypomethylation-mediated activation	EBV miR-BART22; FAM225A and BC200 upregulation	HSP70, sICAM-1, SAA, stathmin, 14-3-3sigma and annexin I upregulation
Immune evasion	*11q13* amplification; Deletions at 9p21.3; Deletions of *TGFBR2*, *TRAF3* and *CYLD*	*ELF3* hypomethylation-mediated activation	EBV miR-BART11 and BART17-3p; miR-106a-5p upregulation	
Therapy resistance		*USP44* hypermethylation-mediated silencing; *FGF5* hypomethylation-mediated activation	RRFERV, PVT1 and HOTAIRM1 upregulation	

## Data Availability

No datasets were generated or analyzed during the current study.
